# Diet as a Therapeutic Measure in Diseases of the Skin

**Published:** 1907-07

**Authors:** 


### DIET AS A THERAPEUTIC MEASURE IN DISEASES OF THE SKIN.

Fox gives some sensible views on this subject and closes with the following:

Finally, I wish to lay stress upon the fact that the character of the food advisable in the treatment of cutaneous and other diseases is of less importance, perhaps, than the manner in which this food is taken. It is not what a patient eats, so much as it is how, and when, and under what circumstances he eats it, that tends to the production of an inflammatory condition of the skin. Hasty eating, irregular eating, and meals taken under the stress of excitement and worry, are the daily experiences of many of our patients both rich and poor. To say that such habits are certain to prove harmful, is merely to utter a platitude, but the assertion that they are often productive of inflammatory skin diseases, is strictly true and worthy of our careful consideration. It is a fact and one which in our reverence for time-worn methods of treatment, and in our enthusiasm over new remedies we are all prone to forget. The subject is a complicated one involving as it does the powerful influence of the nervous system on the nutrition of the skin—and a mere reference to it must suffice.

In discussing diet in the treatment of skin diseases, I can hardly refrain from referring to those two therapeutic handmaidens, exercise and the cold bath. Diet, exercise and bathing! These constitute the tripod of every trainer, restoring health, imparting strength, and inspiring energy. They are the faith, hope and charity of our medical gospel, and just as they tend to put a man in a fit condition for a prize fight or a boat race, just as certainly will they put many of our patients with inflammatory skin disease in a physical condition in which nature alone or assisted by medical skill, will speedily effect a cure. I almost blush to repeat what I have asserted heretofore, that any ignorant trainer can cure many cases of chroni cinflammatory skin diseases, when educated physicians have repeatedly failed, to accomplish this result. And why? Simply because the physician gives good advice which usually the patient does not relish nor heed, and adds a Latin prescription or two, which alone are incapable of curing the disease, but upon which the patient solely relies. The trainer, on the other hand, gives the good advice,—but mark carefully the difference—he also enforces that advice.

In conclusion, let me add that I have no desire to pose as a therapeutic nihilist, decrying the wisdom of the past and all modern therapeutic progress. I am simply raising a feeble voice against routinism in the use of remedies and the common disregard of certain hygienic measures which are simple and sensible—if not highly scientific.—Jour. Cutaneous Diseases, April, '07.

